# Formulation and evaluation of norcanthridin nanoemulsions against the *Plutella xylostella* (Lepidotera: Plutellidae)

**DOI:** 10.1186/s12896-019-0508-8

**Published:** 2019-03-11

**Authors:** Liya Zeng, Yongchang Liu, Jun Pan, Xiaowen Liu

**Affiliations:** Key Laboratory of Comprehensive Utilization of Advantage Plants Resources in Hunan South, College of Chemistry and Bioengineering, Hunan University of Science and Engineering, Yongzhou, Hunan China

**Keywords:** Norcantharidin, Nanoemulsion, Insecticidal, *Plutella xylostella*, Spontaneous emulsification

## Abstract

**Background:**

Norcantharidin (NCTD), a demethylated derivative of cantharidin (defensive toxin of blister beetles), has been reported to exhibit insecticidal activity against various types of agricultural pests. However, NCTD applications are limited by its poor water solubility and high dosage requirement. Nanoemulsions have attracted much attentions due to the transparent or translucence appearance, physical stability, high bioavailability and non-irritant in nature. In general, nanoemulsions with small droplet size can enhance the bioavailability of drugs, whereas this phenomenon is likely system dependent. In present study, NCTD nanoemulsions were developed and optimized to evaluate and improve the insecticidal activity of NCTD against *Plutella xylostella* (Lepidotera: Plutellidae) by a spontaneous emulsification method.

**Results:**

Triacetin, Cremophor EL and butanol were selected as the constituents of NCTD nanoemulsions via solubility determination, emulsification efficiency and ternary phase diagram construction. Insecticidal activity of NCTD nanoemulsion was associated with the content of surfactant and cosurfactant: (1) Higher effective toxicity exhibited at Smix (surfactant to cosurfactant mass ratio) = 3:1 that may be associated with the changes in interfacial tension; (2) NCTD nanoemulsion at 3:7 < SOR (surfactant to oil mass ratio) < 6:4 was more effective at lower surfactant level, which was attributed to the relatively slow diffusion rate of NCTD hindering by excess surfactant. Interestingly, nanoemulsions with smaller droplets were not found to be more effective in our study.

**Conclusions:**

The optimized NCTD nanoemulsion (triacetin/Cremophor EL/butanol (60/20/20, *w*/w)) exhibited effective insecticidal activity (LC_50_ 60.414 mg/l, LC_90_ 185.530 mg/l, 48 h) than the NCTD acetone solution (LC_50_ 175.602 mg/L, LC_90_ 303.050 mg/L, 48 h). Spontaneous emulsifying nanoemulsion employed to formulate this poor water-soluble pesticide is a potential system for agriculture application.

**Electronic supplementary material:**

The online version of this article (10.1186/s12896-019-0508-8) contains supplementary material, which is available to authorized users.

## Background

Cantharidin, the defensive toxin of the blister beetles (Colepotera: Meloidae) and some oedemerid beetles (Colepotera: Oedemeridae) [1], has been reported effective as an anticancer [2–4] and biopesticide [5–7]. However, it is difficult to synthesize cantharidin in the lab and to acquire from natural production. Norcantharidin (NCTD), an available analogue of cantharidin, has been approved for the treatment of multiple types of cancer [8–10]. Both cantharidin and NCTD have been shown to be inhibitor of the highly conserved serine/threonine protein phosphatases (PPs) of eukaryote [11]. In addition, NCTD can act as a natural pesticide because of its effective oral toxicity [12, 13] and strong antifungal activity [14]. Nevertheless, applications of NCTD in agriculture are limited by its poor aqueous solubility and high dosage requirement. Therefore, appropriate formulation delivery systems need to be developed to utilize NCTD in agriculture widely. Formulation is a physical mixture of the one or more biologically active ingredient with a compatible inert (inactive) substances/ filler material in definite proportions. Conventional pesticide and their formulations has raised concerns about their residues in the food and the environment [15–18]. Designing of nanoformulation of different pesticides has newly emerged field for controlling field pests with effective and low environmental risk.

Nanoemulsions-based delivery systems are particularly effective tools for encapsulating lipophilic compounds in pesticides [1, 19, 20]. Nanoemulsions are a class of emulsions with droplet size ranging from 20 to 200 nm and are kinetically stable. Due to their characteristic size, nanoemulsions have been used for improving the bioactivity/bioavailability, stability and safety of some active substances [1, 20, 21]. Many methods have been reported on preparing nanoemulsions for agriculture. Recently, one of the most popular ideas is to prepare nanoemulsions use spontaneous emulsification methods because of its low-cost, simplicity of preparation and without any specialized homogenization equipment [22–24]. However, the main practical problem of spontaneous emulsification that confronts researchers is the requirement of high surfactant or organic solvent concentration [22, 23], which is undesirable on the basis of cost, safety and regulations. Consequently, there is a focus on the potential for NCTD-nanoemulsions that were prepared by the spontaneous emulsification method, stabilized by nonionic surfactant with low content and approved for insecticide use.

It has been proposed that the bioavailability of encapsulated compounds would increase as the droplet size of emulsions decreases, but this is likely to be system dependent. For instance, the antiradical efficiency and antioxidant activity of lycopene nanoemulsions with droplet sizes between 100 and 200 nm were higher than droplet size below 100 nm [21]. That is to say, there are other factors existing affect the bioactivity/bioavailability of the nanoemulsions containing active substances. Most studies undertaken so far have speculated that nanoemulsions increase the lipid solubility of active materials which played an important role in improving bioactive [25]. Moreover, the larger surface area of nano-droplets improves the absorption and digestion of activity components [26]. However, the droplet size fabricated via spontaneous emulsification method is depended upon many factors such as the formulation compound types and content, system composition, interfacial tension, phase behavior, etc. [23, 27, 28]. Therefore, the bioactivity/bioavailability enhancement mechanism of nanoemulsions is still not clear. In particular no study, to our knowledge, has considered the impact of processing conditions, physicochemical characteristics and/or droplet size on insecticidal activity of nanoemulsions formulated by spontaneous emulsification method.

The first objective of this study was to prepare and optimize the NCTD nanoemulsions using the spontaneous emulsification method. Second, the effect of modifying the self-emulsification processing conditions (surfactant and cosurfactant concentration) on the insecticidal activity of NCTD nanoemulsions against the *Plutella xylostella* (Lepidotera: Plutellidae) was investigated. The insecticidal activity of NCTD nanoemulsions with different physicochemical characteristics (droplet size and size distribution) was also evaluated. Particularly, we were interested in investigating the relation between NCTD nanoemulsions characteristics and its bioactivity. The information obtained from this study would provide reference information for designing efficient pesticides for agriculture applications.

## Results

### Solubility determination in oils

NCTD has poor solubility both in the water and oil phase and the maximum solubility of NCTD in water is pH dependent [29]. Table 1 shows the variation of the NCTD saturated solubility across different oils. The highest solubility was observed in triacetin with 12.39 ± 0.21 mg/mL; the lower solubility was exhibited in tributyrin (3.06 ± 0.11 mg/mL). This difference may be attributed to their physical properties such as hydrophilcity, lipophicity or chemical polarity. The minimum solubility (0.44 ± 0.11 mg/mL) was obtained when olive oil was chosen as the oil phase, which could be attributed to its relatively higher viscosity affecting NCTD solubility. Although fatty acid esters have been well represented in many drug delivery systems, ethyl oleate and isopropyl myristate showed poor performance in our study. Numerous previously published studies showed that the addition of medium- and long-chain triglycerides did not reduce the droplet diameter but improved storage stability of nanoemulsions [30, 31]. The lethal concentration (LC_50_) of cantharidin (12.37 mg/L) and NCTD (129.35 mg/L) against *P. xylostella* was reported [32]*.* Accordingly, triacetin was chosen as the optimized oil for the following studies.

**Table 1:** Saturated solubility of NCTD in different oils

Oil Types	Saturated solubility (mg/mL)	Oil Types	Saturated solubility (mg/mL)
Ethyl Oleate	0.72 ± 0.01	Tributyrin	3.06 ± 0.11
Isopropyl Myristate	0.61 ± 0.01	Triacetin	12.38 ± 0.21
Soybean oil	0.47 ± 0.01	MCT	1.23 ± 0.02
Olive oil	0.44 ± 0.01		

Data are expressed as mean ± S.D. (*n* = 3)

### The influence of surfactant

Two types of nonionic surfactants were used to stabilize the nanoemulsions including Cremophor EL, Cremophor RH 40, Tween 20 and Tween 80. The surfactant-to-oil mass ratio (SOR) was held constant at 1:1 and triacetin was used as the oil. The transmittance, droplet size, polydispersity index (PDI) and emulsification time of emulsions formed by spontaneous emulsification were significantly influenced by surfactant types (Table 2). No visible phase separation or creaming was observed in any of the formulation. The smallest droplet size with a bimodal size distribution was formed when Tween 80 was used (*r* = 12.94 ± 0.89 nm, PDI = 0.28 ± 0.07), but fine droplets could also be produced by Cremophor EL (17.03 ± 0.83 nm), Cremophor RH 40 (13.91 ± 0.55 nm) or Tween 20 (40.32 ± 18.08 nm) alone.

**Table 2:** Emulsification ability of various non-ionic surfactants to emulsify the Triacetin (triacetin:surfactant = 1:1, water 96 wt.%, 25 °C)

Surfactant	Molecular weight (g/mol)	HLB	Transmittance (%)	Droplet Size (r.nm)	PDI	Emulsification time (sec)
Cremophor EL	≈ 1630	12–14	100.08 ± 0.02	17.03 ± 0.83	0.33 ± 0.06	82.50 ± 3.12
Cremophor RH 40	≈ 2500	14–16	99.96 ± 0.05	13.91 ± 0.55	0.17 ± 0.08	84.5 ± 6.26
Tween 20	1228	16.7	100.36 ± 0.01	40.32 ± 18.08	0.52 ± 0.14	65.00 ± 7.07
Tween 80	1310	15	99.38 ± 0.03	12.94 ± 0.89	0.28 ± 0.07	171.00 ± 12.00

Data are expressed as mean ± S.D. (*n* = 3)

**Fig. 1:**
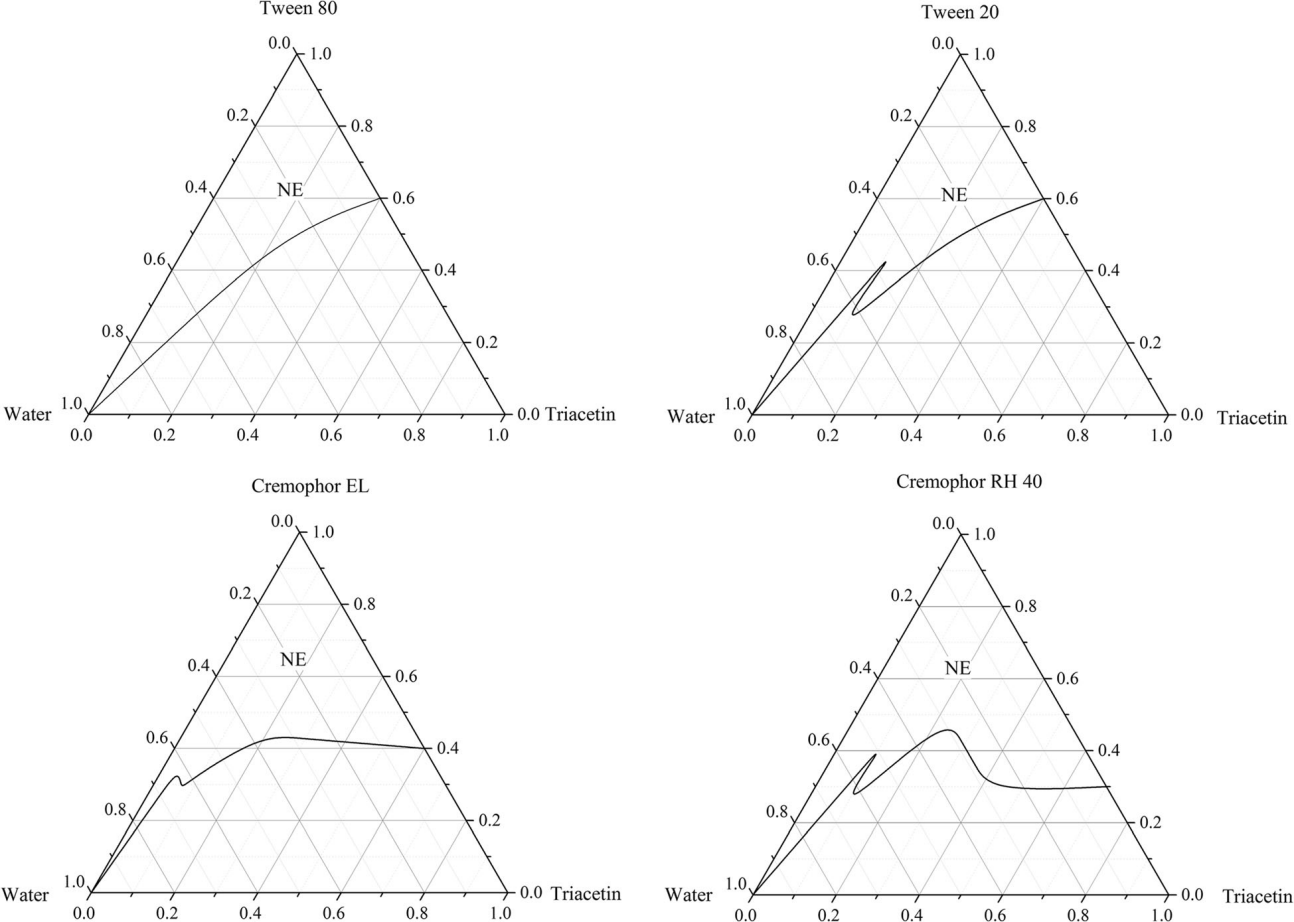
Ternary phase diagrams of water/nonionic surfactant/triacetin systems at 25 °C (NE: nanoemulsion region)

To further understand the emulsification ability of different surfactants, ternary phase diagrams of this three-components system containing water, surfactant and triacetin were constructed. No attempt was made to distinguish between oil-in-water microemulsion, bicontinuous phase, liquid crystalline phase and water-in-oil microemulsions. The oil-in-water nanoemulsions regions are identified in Fig. 1. It was observed that when Tween 20 and Tween 80 were used as surfactants, very low amount of oil (< 40% *w*/w) could be solubilized at a high surfactant concentration (> 60% *w*/w). With further increase the oil concentration to exceed 5:5 (SOR), no nanoemulsion was obtained and phase separation was observed quickly. However, in case of Cremophor EL and Cremophor RH 40, larger nanoemulsion areas were obtained compared to the Tween 20 or Tween 80 system. The maximum amount of triacetin that could be solubilized was increased to 70% w/w. This may indicate that Cremophor have better flexibility and greater ability to reduce the oil-water interfacial tension [33]. Although the nanoemulsions area scale of Cremophor EL formulations was very similar to the Cremophor RH 40, in consideration of size distribution, Cremophor EL was chosen for following study.

### The influence of cosurfactant

The selection of a cosurfactant was executed by preparing a series of emulsions: 50% *w*/w triacetin, Cremophor EL (SOR = 1:1) and cosurfactant (ethanol, ethylene glycol, propanol, isopropanol, propylene glycol, butanol, glycerol, PEG 400, surfactant to cosurfactant mass ratio (Smix) = 3:2).

As shown in Table 3, the formation of nanoemulsions was affected by the addition of cosurfactant: the droplet diameter and particle size distribution were significantly decreased when compared to the cosurfactant-free systems (Table 2). In addition, with the cosurfactant presence, spontaneous emulsification occured conveniently and smaller droplet size formed easily. Similar trends were also observed in relation to the size distribution. The smallest droplets with the narrowest distribution were produced for the system containing butanol (*r* = 6.89 ± 0.126, PDI = 0.04 ± 0.064); the largest droplet size with widest distribution was exhibited for the system with PEG 400 (*r* = 10.45 ± 2.763, PDI = 0.28 ± 0.088).

**Table 3:** Droplet size and size distribution of triacetin/Cremophor EL/cosurfactant/water systems at 25 °C (triacetin:Smix = 1:1, Cremophor EL:cosurfactant = 3:2, water = 96 wt.%)

Cosurfactant	Droplet size (r.nm)	Particle size distribution (PDI)
Ethanol	7.13 ± 0.20	0.09 ± 0.01
Ethylene glycol	7.07 ± 0.11	0.04 ± 0.02
Propanol	7.17 ± 0.12	0.06 ± 0.01
Isopropanol	7.38 ± 0.06	0.08 ± 0.06
Propylene glycol	7.76 ± 0.65	0.09 ± 0.03
Butanol	6.89 ± 0.13	0.04 ± 0.06
Glycerol	7.12 ± 0.12	0.05 ± 0.01
PEG 400	10.45 ± 2.76	0.28 ± 0.09

Data are expressed as mean ± S.D. (*n* = 3)

In order to accurately screen cosurfactants, the transparent temperature range, thermodynamics and dilution stability of nanoemulsions were measured (Additional file 1: Table S1). Most optional systems (except for systems with PEG 400 and ethylene glycol) exhibited thermodynamic and dilution stability. There was no phase separation, creaming or cracking between 4~75 °C in most nanoemulsions. When all of the above problems are considered together, butanol was selected as the cosurfactant to prepare NCTD nanoemulsions.

### The effect of surfactant-to-cosurfactant

**Fig. 2:**
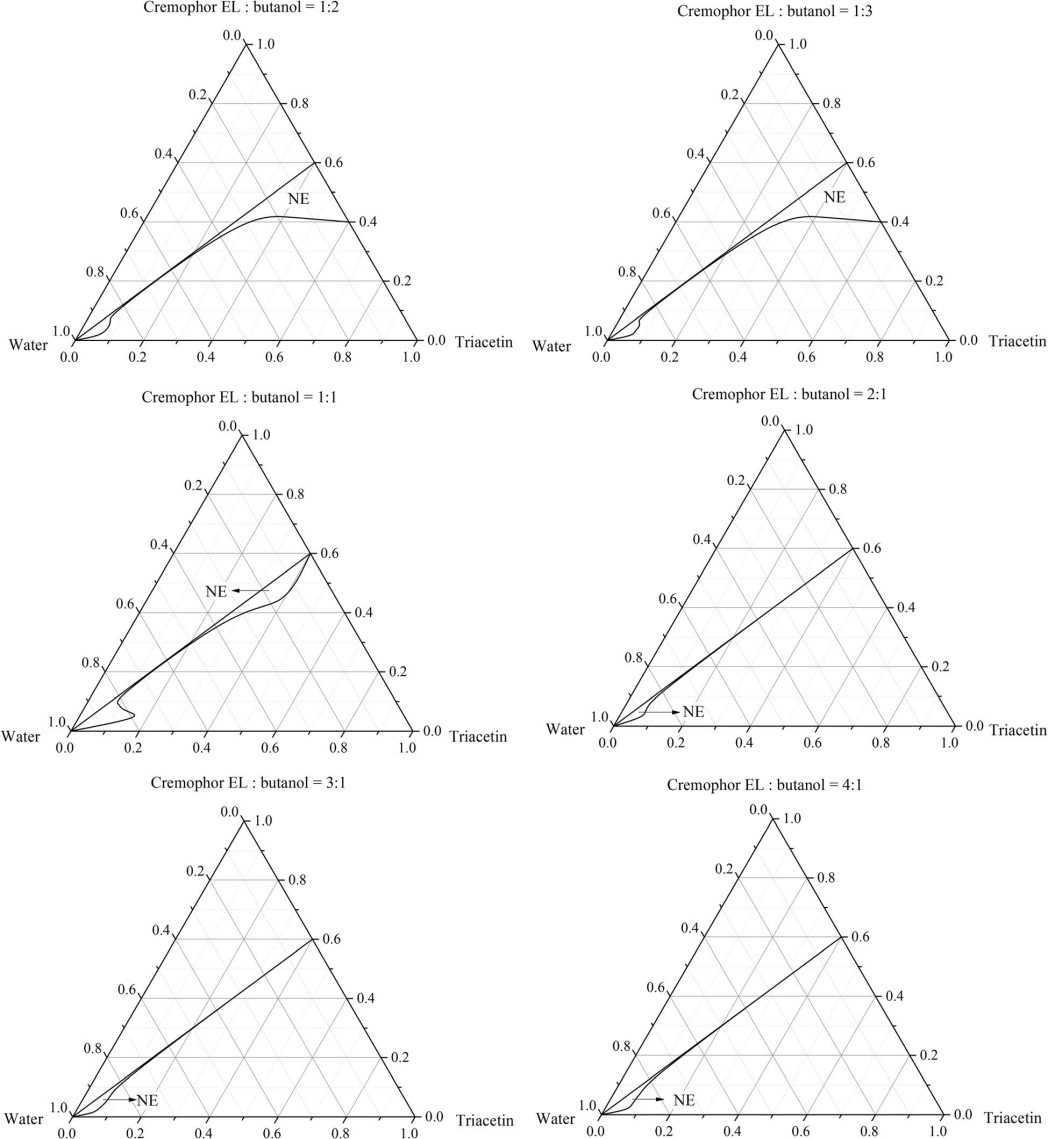
Ternary phase diagrams of water/Cremophor EL-butanol (Smix)/triacetin systems at 25 °C (NE: nanoemulsion region)

To develop a qualified pesticide formulation, the nanoemulsion region in the corresponding ternary phase diagram should be larger to maintain dilution stability and to avoid drug precipitation. Considering the economic efficiency and security, the SOR = 6: 4 was constant in this section. Figure 2 shows the relationship between Smix and the nanoemulsion formation (the NE means the nanoemulsions region, r < 100 nm and PDI < 0.2). The maximum concentration of oil that could be solubilized was 60% *w*/w. When butanol was incorporated along with the Cremophor EL at 1:2 and 1:3, the largest nanoemulsions region was appeared. The smallest nanoemulsions region was observed when the butanol concentration was decreased to Smix = 4:1.

Taken together, the NCTD nanoemulsions containing relatively small droplets and narrow size distribution could be formed with the triacetin/Cremophor EL/butanol/water system. From a commercial perspective, it is advantageous to have high content of triacetin in the nanoemulsions only with low Cremophor EL level were used in the subsequent insecticidal activity studies.

### Insecticidal activity

The insecticidal activity of NCTD and NCTD nanoemulsions (triacetin, Cremophor EL, butanol and 95% *w*/w water) were evaluated. The impact of surfactant and cosurfactant concentration on the insecticidal activity was investigated via the variational Smix (1:3, 1:2, 1:1, 2:1, 3:1 and 4:1) and SOR (3:7, 4:6, 5:5 and 6:4). The insecticidal activity of NCTD-nanoemulsions with different physicochemical characteristics (droplet size and size distribution) was also evaluated.

### NCTD insecticidal activity

Initially, the insecticidal activity of NCTD was determined against third-instar larvae of *P. xylostella*. Results on the mortality treated with increase NCTD concentrations (ranging from 50 to 250 mg/L) and different exposure time are shown in Table 4. Insecticidal activities of NCTD varied according to the exposure time and NCTD concentration. The insecticidal activity of NCTD at 50 mg/L was found to be relatively poor (48 h, mortality rate ≈ 20%), whereas there was 90% mortality at 250 mg/L concentration after 36 h. The fasting larvae rapidly approached to the seedlings with NCTD and fed. No restless movement was observed among the treatment as 90% mortality occured within 48 h. However, the bodies of the poisoned larvae were paralyzed and blacked with diarrhea symptoms. These phenomenons are consistent with earlier results [32, 34], where they investigated the oral toxicities of NCTD against *P. xylostella*. In addition, by increasing the NCTD concentrations (> 200 mg/L), an antifeedant effect was observed and the mortality was remained about the same.

**Table 4:** Oral toxicity of NCTD against the 3rd-instar larvae of *P. xylostella*

Concentration (mg/L)	Mortality rate (%)			
	12 h	24 h	36 h	48 h
CK	0	0	12 ± 8.37	14 ± 8.94
50	0	0	8 ± 13.04	20 ± 15.81
80	0	4 ± 5.48	14 ± 15.17	28 ± 17.89
110	2 ± 4.47	10 ± 7.07	18 ± 8.37	24 ± 11.40
140	8 ± 13.04	12 ± 10.95	26 ± 11.40	26 ± 18.17
170	4 ± 8.94	24 ± 13.42	42 ± 22.80	56 ± 19.49
200	34 ± 34.35	46 ± 25.10	62 ± 24.90	64 ± 26.08
230	28 ± 17.89	48 ± 26.83	72 ± 8.37	72 ± 19.24
250	48 ± 13.04	72 ± 21.68	90 ± 7.07	90 ± 10.00

Data in this table were mean ± SE

The linear regression equation was determined between the NCTD concentration and mortality percentage (Additional file 1: Table S2). Oral toxicities showed that the LC_50_ and LC_90_ values decreased with increasing exposure time, from 247.010 mg/L to 175.602 mg/L and 414.479 mg/L to 303.050 mg/L, respectively. With regard to insecticidal activity, studies conducted by Liu Z [32] showed that NCTD was more active and the *P. xylostella* larvae were killed at lower concentration (LC_50_ = 129.35 mg/L, LC_90_ = 223.29 mg/L). The variation in the toxicity of NCTD against *P. xylostella* may be ascribed to the difference of the susceptible strain and NCTD purification. From the above results, we speculate that NCTD could be applied as a promising biopesticide against *P. xylostella*.

### Effect of cosurfactant concentration

**Fig. 3:**
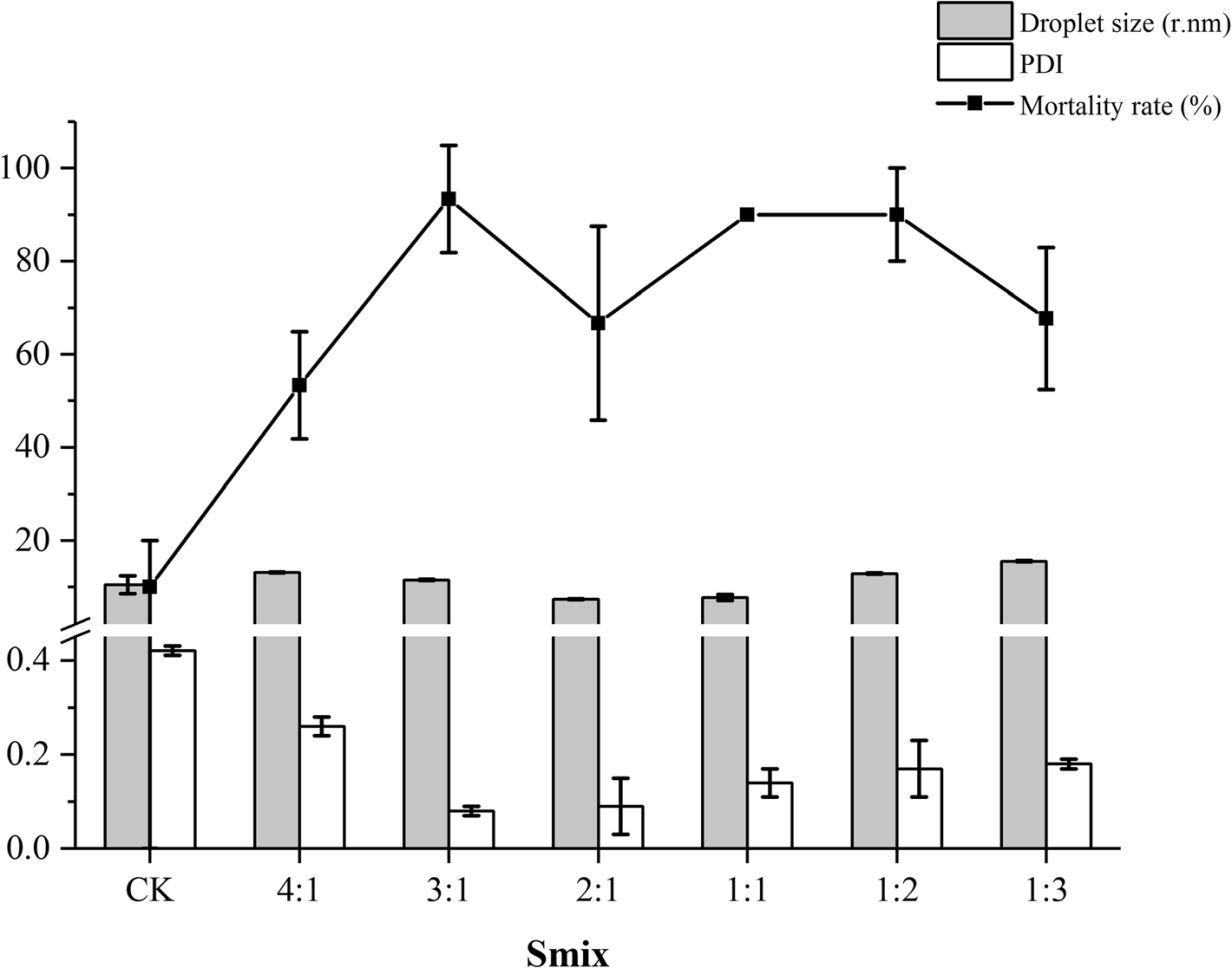
Droplet size, size distribution and mortality rate (48 h) of triacetin/Cremophor EL/butanolt/water systems at 25 °C (triacetin: Smix = 5:5, water = 96 wt.%)

In present investigation, the insecticidal activity of various NCTD nanoemulsions were studied against third-instar *P. xylostella* larvae. In order to better understand the effect of cosurfactant concentration, droplet size and size distribution on the insecticidal activity of NCTD nanoemulsions, the concentration of NCTD was kept constant at 200 mg/L. The effect of butanol concentration on the mortality percentage of NCTD-nanoemulsions stabilized by Cremophor EL (SOR = 1:1) is shown in Table 5 (Additional file 1: Table S3-S5 are corresponded to SOR = 4:6, 6:4 and 3:7). The mortality rate increased with an increase in treatment time. The blank nanoemulsion containing Cremophor EL, triacetin or butanol exhibited week insecticidal activity. Nanoemulsion with an intermediate SOR value 5:5 showed 93.33% mortality at Smix = 3:1 after 48 h; while nanoemulsion formulations at Smix = 4:1 and 2:1, 53 and 67% mortality were observed after 48 h, respectively. To further understand the impact of butanol concentration on the insecticidal activity of NCTD nanoemulsions, the corresponding droplet size and size distribution are shown in Fig. 3. The NCTD nanoemulsion with the smallest droplet size (Smix = 2:1, *r* = 7.38 nm, mortality ≈ 66.67%) was found not to posses the highest mortality after 48 h exposure period.

**Table 5:** Oral toxicity of different NCTD-nanoemulsions (SOR = 4:6, NCTD = 200 mg/L, water = 96% *w*/w) against the 3rd- instar larvae of *P. xylostella*

Smix	Mortality rate (%)			
	12 h	24 h	36 h	48 h
CK	0	10.00 ± 10.00	10.00 ± 10.00	10.00 ± 10.00
4:1	6.67 ± 5.77	23.33 ± 11.55	46.67 ± 15.28	53.33 ± 11.55
3:1	3.33 ± 5.78	53.33 ± 11.55	86.67 ± 11.55	93.33 ± 11.54
2:1	10.00 ± 10.00	36.67 ± 15.28	53.33 ± 15.28	66.67 ± 20.82
1:1	10.00 ± 10.00	46.67 ± 11.55	63.33 ± 15.28	90.00 ± 0
1:2	13.33 ± 5.78	36.67 ± 20.82	60.00 ± 0	90.00 ± 10.00
1:3	16.67 ± 20.82	36.67 ± 20.82	70.00 ± 17.32	76.67 ± 15.28

Data in this table were mean ± SE

### Effect of surfactant concentration

**Fig. 4:**
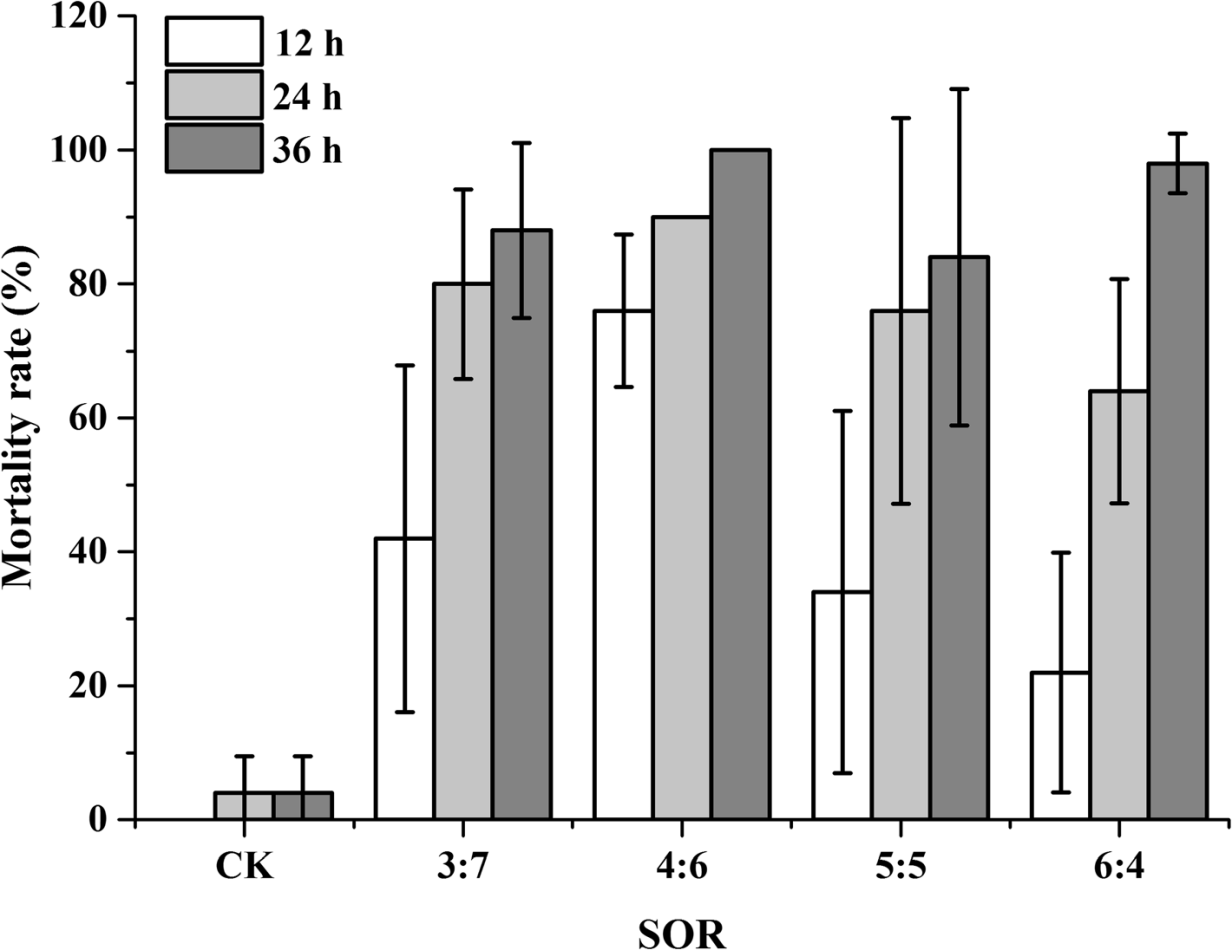
The effect of different surfactant-to-oil mass ratio (SOR) on the oral toxicity of NCTD-nanoemulsion (Smix = 1:1, NCTD 200 mg/L) against the third-instar of *P. xylostella*

In this section, we examined the influence of surfactant concentration on the insecticidal activity of NCTD nanoemulsions (Smix =1:1 and NCTD = 200 mg/L). As can be seen in Fig. 4, the higher mortality was observed at lower SOR in the first 12 h where larger droplets were formed [24, 31, 35]; a similar trend was observed in the next 12 h. The highest mortality (100%) was obtained from the nanoemulsions with SOR 4:6 after 36 h and a slight lower mortality (98%) was exhibited at SOR = 6:4 under the same time. Nanoemulsions with smaller droplets having better insecticidal activity was also not found in this experiment.

### Optimized NCTD-nanoemulsion

On the basis of Section 3.5.2 and 3.5.3, the optimized NCTD-nanoemulsion formulation was consisted of triacetin/Cremophor EL/butanol (60/20/20, *w*/w) and the LC_50_ and LC_90_ of this formulation were determined in Table 6. A positive linear correlation was observed between the NCTD concentration and mortality, and the LC_50_ and LC_90_ value decreased as the exposure time increased. The LC_50_ (60.414 mg/l, 48 h) or LC_90_ (185.530 mg/l, 48 h) value of the optimizing formulation indicates that NCTD encapsulated in the nanoemulsion was more toxic than NCTD acetone solution (Additional file 1: Table S2, LC_50_ = 175.602 mg/L, LC_90_ = 303.050 mg/L, 48 h).

**Table 6:** Regression equation of NCTD against 3rd-instar larvae of *P. xylostella*

Time	Regression equation	R^2^	χ^2^	LC_50_ (mg/L) (95% confidence limits)	LC_90_ (mg/L) (95% confidence limits)
12 h	–	–	–	–	–
24 h	*y* = 6.379*x* - 14.881	0.921	0.462	150.090 (114.135~310.777)	312.188 (203.841~10,200.727)
36 h	*y* = 5.637*x* - 12.727	0.992	0.156	75.992 (30.489~108.489)	191.903 (130.619~791.051)
48 h	*y* = 2.630*x* – 4.684	0.985	0.264	60.414 (23.136~92.727)	185.530 (117.860~693.284)

LC_50_ = Lethal concentration at which 50% of the larvae showed mortality

LC_90_ = Lethal concentration at which 90% of the larvae showed mortality

*x* = log concentration

*y* = percentage mortality

## Discussion

The spontaneous emulsification method was used to fabricate nanoemulsions by adding an oil and hydrophilic surfactant mixture into the aqueous phase [35]. Studies have demonstrated that the rapid movement of hydrophilic surfactant from oil to aqueous phase is one of the most significant determinants affecting the formation of oil droplets by spontaneous emulsification [24, 28, 35]. Our transmittance values were not in accordance with earlier results [36], according to which the transmittance increased with decreasing droplet size. This effect can be attributed to the droplets multimodal distribution: the bigger droplets scattered light strongly though the formulation had a translucent appearance. The affinity of surfactant for the hydrophobic or hydrophilic phase is a significantly important factor for the nanoemulsions formation [24, 35]. The HLB value of four surfactants were around 15 in our study while, different emulsification abilities were exhibited. That indicates that there were other factors responsible for spontaneous emulsification, such as the molecular geometry of surfactant.

It is well known that the main drawback of spontaneous emulsification is the requirement of relatively high amounts of synthetics surfactants to maintain stability. Consequently, there is interest in developing stable nanoemulsions with small droplets and low surfactant concentration. Cosurfactant addition is an appealing idea for assisting a surfactant in reducing interfacial tension, fluidifying interfacial film, controlling droplet size and improving nanoemulsion stability [37, 38]. What is notewothy is that the smallest droplets with the narrowest distribution were produced for the system containing butanol, whilt the largest droplet size with widest distribution was exhibited with PEG 400. This phenomenon was agreed with our previous hypothesis postulating that the oil-water interfacial may be more flexible during spontaneous emulsification in cosurfactant-presence system, due to the hydrophilicity and viscosity of cosurfactant [33]. It is worth noting that some cosurfactants are volatile, a factor making it impractical for transportation and storage.

The concentration of the butanol had a notable impact on the spontaneous emulsification of emulsions. Additionally, spontaneous emulsification became easier and faster which was attributed to the combination of Cremophor EL and butanol decreased the oil-water interfacial tension and increased the flexibility of the surfactant layer. Decreasing the butanol content, the nanoemulsions areas were reduced appreciably. However, the opposite observation was found in previous report where the nanomeulsion region was increased with decreased cosurfactant concentration [38]. This difference could be explained by the effect of triacetin that acted as an emulsifier and co-emulsifier simultaneously in our systems.

In this study, the cosurfactant concentration significantly influenced the insecticidal activity of NCTD nanoemulsions prepared by spontaneous emulsification; this has not yet been reported, and the mortality was in the following order: 3:1 > 1:1 = 1:2 > 1:3 > 2:1 > 4:1(48 h). As above, the mortality rate of NCTD-nanoemulsions in the first 24 h exhibited a similar order. This might be associated with the presence of 5–10% *w*/w alcohol which could give rise to a change in the interfacial tension [39, 40]. In addition, there was a nonlinear correlation between droplet size or size distribution and the mortality rate of *P. xylostella*. This finding does not agree with previous research indicating that nanoemulsions with smaller droplet size were found to be more effective in controlling pests compared with larger droplet sizes [1, 20, 41]. In consequence, our study highlights that there are other factors such as cosurfactant concentration that need to be considered.

The mortality rate of NCTD nanoemulsions was obviously dependent on surfactant concentration and exposure time. The higher mortality of NCTD nanoemulsions was observed at lower SOR, coinciding with the finding of a previous study that the release of drug was relatively slow due to the protection of droplets structure by sufficient surfactant [25]. However, the highest mortality was obtained from the nanoemulsions with SOR 4:6. This phenomenon may be related to the appearance of micelles, attributed to the excess surfactant, which hinders the NCTD release [42, 43].

Compared the insecticidal activity of optimized NCTD nanoemulsion with the NCTD, the NCTD nanoemulsion was found to be more effective than its acetone solution. This finding was in agreement with many previous showed that nanoparticles can improve the bioactivity or bioaccessibility of active materials [1, 20, 21]. A pesticide with a lower LC_50_ indicates it takes less amount of the pesticide to kill half of the test insects; therefore nanoemulsions may be a good choice as a potent and selective delivery system for pesticides.

## Conclusion

In this investigation, nanoemulsion containing poorly water-soluble NCTD was optimized and prepared using spontaneous emulsification method to prevent the *P. xylostella* larvae. The compositions for the NCTD-nanoemulsion were selected by a solubility study, emulsification ability analysis and ternary phase diagrams construction. The surfactant and cosurfactant concentration significantly impacted the insecticidal activity of NCTD nanoemulsions. Surfactant concentration notably affects the oil-water interface structure and hinders the drug release from nano-droplets. However, nanoemulsions containing smaller droplets had better oral toxicity was not found in our study. In consequence, evaluation of the relationship between exposure time and drug release mechanism of nanoemulsions is a promising orientation for our further study. The composition of NCTD-nanoemulsion was optimized as 60% triacetin, 20% Cremophor EL and 20% butanol, which showed a higher efficacy as an insecticidal agent against *P. xylostella* when compared to NCTD acetone solution. It can be speculated that nanoemulsions may be a good option to other pesticides for the agriculture applications.

## Methods

### Materials

Norcantharidin (NCTD) was obtained from Alfa Aesar (> 98%, Ward Hill, MA). Soybean oil, olive oil, ethyl oleate, isopropyl myristate, triacetin, tributyrin, Tween 80, Tween 20, ethanol, ethylene glycol, 1-propanol, isopropanol, propylene glycol, 1-butanol, glycerol and PEG 400 were purchased from Aladdin (> 98%, Shanghai, China). Medium chain triglyceride (MCT), Cremophor EL and Cremophor RH 40 were obtained from BASF (Ludwigshafen, Germany). All other chemicals and solvent were of analytical grade and used without further purification.

Deionized water was generated from the Milli-Q gradient system of Millipore (Synergy, Millipore SAS, Molsheim, France).

### Selection of nanoemulsions composition

The oil was determined by the saturation solubility of NCTD using shake flask method [44]. Excess amount of NCTD was added into each vial with various oils under vortex mixing for 30s. Then, mixtures were incubated in a shaking table at constant temperature (37 ± 3 °C, 50 strokes/min) to reach equilibrium states. After 3 days, mixtures were centrifuged (5000 rpm, 5 min) and the supernatants were filtered through a Millipore membrane filter (0.45 μL). The quantify analysis was implemented by the Agilent Gas Chromatograph [10, 42].

Surfactants and co-surfactants were screened for their emulsification ability according to the published research [44] with slight modification. Briefly, oil (500 mg) was homogenized with surfactant (500 mg) followed by warming at 50 °C for 30 s. Various isotropic mixtures (200 mg) were diluted to 50 mL deionized water to yield fine emulsions. Emulsification time was employed to evaluate the emulsification ability of different surfactants. Similarly, screening of co-surfactants was implemented using the same method as described above. The selected surfactant (300 mg) was mixed with different co-surfactant (200 mg) and then blended with optimal oil (500 mg).

### Construction of ternary phase diagrams

The nanoemulsions region was determined using ternary phase diagrams. Each corner corresponded to 100% of water, surfactant and oil. Various compositions were prepared by altering the water concentration in the order of 5% while the amount of surfactant and oil remained fixed. The homogeneous mixtures were then diluted with distilled water and evaluated transmittance, droplet size and polydispersity index (PDI). The mixtures formed emulsions with droplet size < 100 nm and PDI < 0.2 were considered to be in the nanoemulsions region.

### Preparation of nanoemulsions

Nanoemulsions were prepared by spontaneous emulsification method [22] with slight modification. Spontaneous emulsification was performed by adding the oil phase into and aqueous phase under mildly stirring (500 rpm, 15 min) at room temperature (25 ± 2 °C). The oil phase was composed of oil, surfactant and co-surfactant.

### Droplet size and size distribution

The droplet size and PDI were determined by dynamic light scattering (Nano ZS, Malvern Instrument Ltd., Worcestershire, UK) at a fixed scattering angle of 173° and with the laser wavelength of 633 nm. Measurements were conducted at 25 °C and each measurement was made with three readings per sample. In addition, measurements were carried out for all samples after equilibrating for 2 h.

### Thermodynamic stability study

The thermodynamic stability of formulated nanoemulsions were subjected to the following stresses: (1) Centrifugation: the formulated nanoemulsions were centrifuged at 5000 rpm for 30 min and observed for phase separation, creaming or cracking. (2) Heating-cooling cycle: formulated nanoemulsions, after centrifugation tests, were stored at refrigerated temperature of 4 °C and 40 °C for 48 h respectively. This cycle was repeated six times. (3) Freeze-thaw cycle: formulated nanoemulsions were kept at − 20 °C and 25 °C for 48 h at each temperature. The cycle was repeated three times.

### Insecticidal activity

*Plutella xylostella* (Lepidotera: Plutellidae) was chosen as the test insect. It is a worldwide pest of cruciferous vegetables and an important focus of research. The susceptible strain was obtained from the Key Lab of Plant Protection Resources & Pest Management of Ministry of Education, Northwest A&F University (Yangling, China). The larvae were fed with pakchoi (*Brassica chinensis* L.) (25 ± 2 °C, RH 50 ± 5%, L: D = 16:8).

Third-instar larvae of *P. xylostella* were randomly chosen and separated in three treatment groups. One group was topically applied with nanoemulsions contain NCTD, while negative control groups were treated with blank nanoemulsions and NCTD solution (NCTD was dissolved in mixed solvent (water: acetone: dimethyl sulfoxide = 20: 19: 1, *V*/V) with 0.5% Tween 80 to different concentration) respectively. The bioactivities of all groups were tested using seedlings of pakchoi (without roots). For all test groups, the seedlings were dipped for 5 s, then placed on a blotting paper to drain superfluous fluid and left to dry in the room temperature.

Ten treated seedlings were put in the Petri plate (9 cm diametr) with wetting filter paper to keep them fresh. Larvae were released on the plate after fasting for 3 h and then kept in growth cabinet (25 ± 2 °C, RH 50 ± 5%, L: D = 16:8). All experiments were repeated at least five replicates with samples from 10 larvae. Percentage mortalities were determined 24 and 48 h post-treatment. Test insects were considered dead when, prodded with fine small brush (maximum three times), they showed no appendage movement.

### Statistical analysis

All statistical test data were expressed as the mean ± standard deviation (SD). The LC_50_ value and the associated 95% confidence interval (*P* < 0.05) was determined using the Probit method [12]. The observed mortalities and effluent concentrations were transformed into a probit transformation and log_10_, respectively. This involves estimation of linear regression parameters by an interactive approach. The LC 50 and associated confidence interval were calculated from the estimated linear regression parameters.

## Additional file

Additional file 1: **Table S1.** The performance of nanoemulsions with different cosurfactants. **Table S2.** Regression equation of NCTD against the 3rd-instar larvae of *P. xylostella.* **Table S3.** Oral toxicity of different surfactant-to-cosurfactant mass ratio (Smix) against the 3rd- instar larvae of *P. xylostella* (SOR =4:6). **Table S4.** Oral toxicity of different surfactant-to-cosurfactant mass ratio (Smix) against the 3rd- instar larvae of *P. xylostella* (SOR = 6:4). **Table S5.** Oral toxicity of different surfactant-to-cosurfactant mass ratio (Smix) against the 3rd- instar larvae of *P. xylostella* (SOR = 3:7). Figures.xlsx Sheet1 Fig. 1 Ternary phase diagrams of water/nonionic surfactant/triacetin systems at 25 °C (NE: nanoemulsion region). Figures.xlsx Sheet2 Fig. 2 Ternary phase diagrams of water/Cremophor EL-butanol (Smix)/triacetin systems at 25 °C (NE: nanoemulsion region). Figures.xlsx Sheet3 Fig. 3 Droplet size, size distribution and mortality rate (48 h) of triacetin/Cremophor EL/butanolt/water systems at 25 °C (triacetin: Smix = 5:5, water = 96 wt.%). Figures.xlsx Sheet4 Fig. 4 The effect of different surfactant-to-oil mass ratio (SOR) on the oral toxicity of NCTD-nanoemulsion (Smix = 1:1, NCTD 200 mg/L) against the third-instar of *P. xylostella*. (ZIP 31 kb)

## Abbreviations

NCTD: Norcantharidin
NE: Nanoemulsion region
PPs: Serine/threonine protein phosphatases
Smix: Surfactant-to-cosurfactant mass ratio
SOR: Surfactant-to-oil mass ratio
