# Supplementary material for: Formulation and evaluation of norcanthridin nanoemulsions against the Plutella xylostella (Lepidotera: Plutellidae)
Source: BMC Biotechnol. 2019 Mar 11;19:16. doi: 10.1186/s12896-019-0508-8 (PMC6419361; doi:10.1186/s12896-019-0508-8)
Supplement: Supplementary file 1 — Table S1. The performance of nanoemulsions with different cosurfactants. Table S2. Regression equation of NCTD against the 3rd-instar larvae of P. xylostella. Table S3. Oral toxicity of different surfactant-to-cosurfactant mass ratio (Smix) against the 3rd- instar larvae of P. xylostella (SOR =4:6). Table S4. Oral toxicity of different surfactant-to-cosurfactant mass ratio (Smix) against the 3rd- instar larvae of P. xylostella (SOR = 6:4). Table S5. Oral toxicity of different surfactant-to-cosurfactant mass ratio (Smix) against the 3rd- instar larvae of P. xylostella (SOR = 3:7). Figures.xlsx Sheet1 Fig. 1 Ternary phase diagrams of water/nonionic surfactant/triacetin systems at 25 °C (NE: nanoemulsion region). Figures.xlsx Sheet2 Fig. 2 Ternary phase diagrams of water/Cremophor EL-butanol (Smix)/triacetin systems at 25 °C (NE: nanoemulsion region). Figures.xlsx Sheet3 Fig. 3 Droplet size, size distribution and mortality rate (48 h) of triacetin/Cremophor EL/butanolt/water systems at 25 °C (triacetin: Smix = 5:5, water = 96 wt.%). Figures.xlsx Sheet4 Fig. 4 The effect of different surfactant-to-oil mass ratio (SOR) on the oral toxicity of NCTD-nanoemulsion (Smix = 1:1, NCTD 200 mg/L) against the third-instar of P. xylostella. (ZIP 31 kb) [file 12896_2019_508_MOESM1_ESM.zip › Table S.docx]

Table S1 The performance of nanoemulsions with different cosurfactants

| Cosurfactant | Visual observation | Low-temperature stability | Heat stability | Dilution stability | Range of transparent (℃) |
| --- | --- | --- | --- | --- | --- |
| Ethanol | transparent | pass | pass | pass | 4~75 |
| Ethylene glycol | turbidity | fail | fail | pass | 4~75 |
| Propanol | transparent | pass | pass | pass | 4~75 |
| Isopropanol | transparent | pass | pass | pass | 4~75 |
| Propylene glycol | transparent | pass | pass | pass | 4~75 |
| Butanol | transparent | pass | pass | pass | 4~70 |
| Glycerol | transparent | pass | pass | pass | 4~75 |
| PEG 400 | turbidity | fail | fail | pass | 4~75 |

Table S2 Regression equation of NCTD against the 3^rd^-instar larvae of *P. xylostella*

| Time | Regression equation | R^2^ | χ^2^ | LC_50_ (mg/L)  (95% confidence limits) | LC_90_ (mg/L)  (95% confidence limits) |
| --- | --- | --- | --- | --- | --- |
| 12 h | *y* = 5.701*x* - 13.641 | 0.992 | 0.144 | 247.010  (202.890~466.588) | 414.479  (293.079~2266.063) |
| 24 h | *y* = 6.379*x* - 14.881 | 0.971 | 0.647 | 215.204  (188.947~267.802) | 341.782  (272.588~665.794) |
| 36 h | *y* = 5.637*x* - 12.727 | 0.954 | 1.329 | 181.065  (152.206~214.894) | 305.622  (247.257~518.536) |
| 48 h | *y* = 5.408*x* – 12.138 | 0.969 | 0.942 | 175.602  (131.483~207.485) | 303.050  (241.959~804.467) |

LC_50_ = Lethal concentration at which 50% of the larvae showed mortality.

LC_90_ = Lethal concentration at which 90% of the larvae showed mortality.

*x* = log concentration

*y* = percentage mortality

Table S3 Oral toxicity of different surfactant-to-cosurfactant mass ratio (Smix) against the 3^rd^- instar larvae of *P. xylostella* （SOR =4:6）

| Smix | Mortality rate (%) | | | |
| --- | --- | --- | --- | --- |
|  | 12h | 24 h | 36 h | 48 h |
| CK | 0 | 0 | 4.33 ± 5.13 | 13.33 ± 15.28 |
| 4:1 | 6.67 ± 11.55 | 13.33 ± 15.28 | 63.33 ± 15.28 | 70.00 ± 10.00 |
| 3:1 | 3.33 ± 5.77 | 20.00 ± 17.32 | 50.00 ± 0 | 53.33 ± 5.78 |
| 2:1 | 6.67 ± 5.77 | 23.33 ± 11.55 | 33.33 ± 5.77 | 36.67 ± 5.78 |
| 1:1 | 10.00 ± 17.32 | 30.00 ± 10.00 | 46.67 ± 32.15 | 73.33 ± 15.28 |
| 1:2 | 16.67 ± 28.82 | 40.00 ± 45.83 | 66.67 ± 25.17 | 70.00 ± 26.46 |
| 1:3 | 16.67 ± 20.82 | 36.67 ± 15.28 | 73.33 ± 5.78 | 83.33 ± 15.28 |

Data in this table were mean ± SE.

Table S4 Oral toxicity of different surfactant-to-cosurfactant mass ratio (Smix) against the 3^rd^- instar larvae of *P. xylostella* （SOR = 6:4）

| Smix | Mortality rate (%) | | | |
| --- | --- | --- | --- | --- |
|  | 12h | 24 h | 36 h | 48 h |
| CK | 0 | 0 | 0 | 0 |
| 4:1 | 13.33 ± 15.28 | 43.33 ± 11.55 | 63.33 ± 15.28 | 76.67 ± 5.77 |
| 3:1 | 16.67 ± 20.82 | 66.67 ± 25.17 | 86.67 ± 11.55 | 90.00 ± 10.00 |
| 2:1 | 13.33 ± 5.77 | 50.00 ± 10.00 | 73.33 ± 15.28 | 86.67 ± 5.77 |
| 1:1 | 30.00 ± 10.00 | 70.00 ± 17.32 | 80.00 ± 10.00 | 96.67 ± 5.77 |
| 1:2 | 16.67 ± 11.55 | 46.67 ± 20.82 | 60.00 ± 20.00 | 66.67 ± 25.17 |
| 1:3 | 0 | 20.00 ± 10.00 | 43.33 ± 15.28 | 43.33 ± 15.28 |

Data in this table were mean ± SE.

Table S5 Oral toxicity of different surfactant-to-cosurfactant mass ratio (Smix) against the 3^rd^- instar larvae of *P. xylostella* （SOR = 3:7）

| Smix | Mortality rate (%) | | | |
| --- | --- | --- | --- | --- |
|  | 12h | 24 h | 36 h | 48 h |
| CK | 0 | 3.33 ± 5.77 | 10.00 ± 10.00 | 10.00 ± 10.00 |
| 4:1 | 23.33 ± 20.82 | 60.00 ± 36.06 | 86.67 ± 23.09 | 100.00 |
| 3:1 | 3.33 ± 5.78 | 23.33 ± 15.28 | 83.33 ± 5.78 | 86.67 ± 5.77 |
| 2:1 | 0 | 20.00 ± 17.32 | 40.00 ± 26.46 | 90.00 ± 10.00 |
| 1:1 | 13.33 ± 11.55 | 53.33 ± 15.28 | 90.00 ± 0 | 93.33 ± 5.77 |
| 1:2 | 6.67 ± 11.55 | 13.33 ± 5.77 | 50.00 ± 0 | 76.67 ± 20.82 |
| 1:3 | 16.67 ± 15.28 | 50.00 ± 20.00 | 83.33 ± 5.78 | 83.33 ± 15.28 |

Data in this table were mean ± SE.
